# Correlates of susceptibility to waterpipe tobacco smoking in young adults

**DOI:** 10.1371/journal.pone.0307058

**Published:** 2024-07-16

**Authors:** Mahmood A. Alalwan, Lauren Long, Elise M. Stevens, Brittney Keller-Hamilton, Andrea C. Villanti, Glenn Leshner, Theodore L. Wagener, Darren Mays

**Affiliations:** 1 Department of Internal Medicine, The Ohio State University College of Medicine, Center for Tobacco Research, The Ohio State University Comprehensive Cancer Center, Columbus, Ohio, United States of America; 2 Division of Preventive and Behavioral Medicine, Department of Population and Quantitative Health Sciences, University of Massachusetts Chan Medical School, Worcester, Massachusetts, United States of America; 3 Department of Health, Behavior, Society, and Policy, Rutgers School of Public Health, Rutgers Institute for Nicotine & Tobacco Studies, New Brunswick, New Jersey, United States of America; 4 Gaylord College of Journalism and Mass Communication, University of Oklahoma, Norman, Oklahoma, United States of America; UT Austin: The University of Texas at Austin, UNITED STATES OF AMERICA

## Abstract

**Introduction:**

Many US young adults are susceptible to waterpipe (i.e., hookah) tobacco smoking (WTS) initiation, but research on factors associated with WTS susceptibility is limited. We examined sociodemographic, other tobacco and substance use, and attitudes and perceptions correlates of WTS susceptibility among young adults.

**Methods:**

Baseline data from a randomized trial testing WTS risk messages was collected in US young adults aged 18 to 30 years who never used waterpipe tobacco but were susceptible to WTS (n = 294). Extent of susceptibility to WTS was defined using the average score of a valid scale with higher scores indicating higher susceptibility. Correlates were sociodemographics, other tobacco and substance use, and attitudes and perceptions. Multiple linear regression models identified correlates of greater WTS susceptibility.

**Results:**

Participants averaged 25 (SD 3.2) years of age, 60% were male, 22% were Black non-Hispanic, 47% completed some college education, and 66% were employed. Our models consistently showed that more positive attitudes toward WTS (*β* = -0.08, *p*<0.01), lower perceived addictiveness relative to cigarettes (*β* = -0.09, *p* = 0.04), and greater perceived social acceptability of WTS (*β* = 0.05, *p*<0.01) were positively correlated with WTS susceptibility. Additionally, young adults who smoked cigarillos (*β* = 0.53, *p*<0.01), used cannabis (*β* = 0.14, *p* = 0.02), and Black non-Hispanic versus White non-Hispanic young adults (*β* = 0.18, *p* = 0.03) also had higher WTS susceptibility.

**Conclusions:**

Findings suggest that WTS prevention efforts require multicomponent interventions including targeting subpopulations at greater risk based on race/ethnicity and other tobacco and substance use. These interventions should consider attitudes and social acceptability of WTS as modifiable targets to maximize public health benefits.

## Introduction

Waterpipe (also known as hookah, narghile, or shisha) tobacco smoking (WTS) exposes users to toxicants and poses similar health risks to cigarette smoking, including cancer and pulmonary and cardiac diseases [1, 2]. In the United States (U.S.), the prevalence of past 30-day of WTS is highest among young adults aged 18 to 30 years [3]. For instance, according to the latest data publicly available from the Population Assessment of Tobacco and Health (PATH) survey (Wave 5, 2019), past 30-day use of WTS was highest in young adults (5.1%), compared to youth (0.3%) and older adults (1.4%) [3]. Over time, many U.S. young adults continue to use waterpipe tobacco, albeit in use patterns that differ from other combustible tobacco products such as cigarettes (e.g., monthly rather than daily) [4].

This high prevalence of WTS among young adults is attributable to several factors, including demographic characteristics such as younger age and being male [4–7]. Additionally, other factors contributing the high prevalence of WTS in this population include potentially modifiable factors, which refer to elements or perceptions that can be influenced or shaped to reduce the risk of WTS, such as lower perceived risks of and more positive attitudes towards WTS [8–13]. WTS is also facilitated by permissive social norms surrounding WTS, use in social settings (e.g., lounges, bars), and availability of sweetened and flavored waterpipe tobacco [14, 15]. For these reasons, WTS has been characterized as a “starter product,” or a tobacco product that appeals to young people in particular [16]. Existing research has consistently described factors related to initiation and cessation of WTS [8–15, 17]. However, little is known about the factors that contribute to WTS susceptibility, which is an important upstream factor for behavioral interventions due to young adults’ higher risk and susceptibility to WTS.

In young adults, unique psychological and biological factors set them apart from both adolescents and older adults, such as emotional distress and shifts in social and environmental factors associated with the transition to autonomy [18]. These factors increase the vulnerability of young adults to experimentation and persistence in tobacco use. Given their higher risk and susceptibility to WTS, young adults who do not smoke waterpipe are a key population to target preventive efforts. Susceptibility—a construct that relies on strong theoretical underpinnings (i.e., the association between intention and behavior)—to WTS is defined as the lack of a firm decision against use. Evidence from cross-sectional and longitudinal studies indicate that susceptibility to WTS is an important predictor of initiation among never-user young adults as well as continued use among ever-users [19–22]. For instance, a study among college students reported that 27% were susceptible to WTS. Among them, 20% had tried WTS a year later [21]. In another nationally representative sample of young adults aged 18 to 30 years, researchers reported 22% were susceptible to WTS. Those who were susceptible to WTS had nearly seven times greater odds of initiating WTS after 18 months compared to those who were not susceptible [12]. Several reviews show the scarcity of interventions to prevent WTS, with only a few interventions among young adult users and those who do not smoke waterpipe but are susceptible to WTS, a population at higher risk of WTS initiation [20, 23, 24]. These interventions were mainly risk-based messages designed to counter misperceptions about health and addiction risks associated with WTS [9, 13, 25–29].

There is a limited number of interventions to date to prevent initiation of WTS, which may be due in part to lack of clarity on what populations or factors to target. For example, few studies examined demographic (e.g., race-ethnicity) and behavioral (e.g., substance use) factors to identify subgroups at greater susceptibility to WTS among young adults [20]. In particular, young adults are more susceptible to poly-tobacco use and experimentation with emerging tobacco products [30, 31]. They are likely to be concurrent users of waterpipe, cigarillos, little cigars, and electronic cigarettes (e-cigarettes) [32–34]. Additionally, there is little research on potentially modifiable factors, such as attitudes, perceptions, and social norms, associated with susceptibility to WTS [20, 35]. Identifying these factors can guide the development of new intervention strategies and refinement of available interventions to reduce the WTS burden among young adults. This study sought to address this gap and advance our understanding of young adults’ susceptibility to WTS by examining sociodemographic, behavioral (other tobacco and substance use), and attitudes and perceptions associated with WTS susceptibility. To achieve this objective, we used a series of models that examined potentially modifiable factors with and without demographic and behavioral factors.

## Methods

### Participants and procedures

This is a secondary analysis of baseline data from a randomized controlled trial (NCT04252014) testing the effects of messages communicating health and addiction risks of WTS [35]. Participants for this analysis were young adults aged 18 to 30 years, able to complete study procedures online in English, and had never smoked waterpipe tobacco but were susceptible to initiating WTS based on responses to a valid, 4-item susceptibility measure [20]. Participants were recruited using a nationally representative consumer research panel (National Opinion Research Council (NORC) AmeriSpeak) between February 4, 2020 and March 3, 2020. Using probability-based sampling, NORC recruits AmeriSpeak panel members using the NORC National Frame and address-based sampling frames [36]. Participants completed a baseline assessment online with measures of WTS and baseline trial outcomes. All eligible participants provided online informed consent to complete enrollment, and the participating institutions’ institutional review boards approved procedures (2020C0080).

### Measures

#### Dependent variable

*Extent of susceptibility to WTS*. We used the average score of four valid susceptibility items (Cronbach α = 0.84) [20]. These four items were: 1) Do you think that you will smoke tobacco from a waterpipe soon? 2) Do you think that you will smoke tobacco from a waterpipe in the next year? 3) Do you think that in the future you might experiment with waterpipe tobacco smoking? 4) If one of your best friends asked you to smoke tobacco from a waterpipe, would you? Response options were on a scale of 1 (Definitely Yes) to 4 (Definitely No). Those who responded “Definitely No” to all items were considered non-susceptible. We reversed the scale for these four items with higher scores indicating higher susceptibility to WTS.

#### Independent variables

*Sociodemographics*. Demographic characteristics from participants’ AmeriSpeak profiles included age, sex (male/female), race (Black/Asian/Others), ethnicity (Hispanic/Non-Hispanic), household income (Range from 1 (Less than $5,000) to 18 ($200,000 or more)), educational attainment (High school or less/Some college/Bachelor’s degree or higher), employment status (Yes/No), and marital status (Yes/No).

*Other tobacco and substance use*. We assessed other tobacco product and substance use including past-30-day (Yes/No) cigarette, cigar, cigarillo, smokeless tobacco, e-cigarette, alcohol, and cannabis use.

*Attitudes towards WTS*. We measured attitudes towards WTS by averaging responses to four pairs (Positive–Negative, Good–Bad, Like–Dislike, and Pleasant–Unpleasant) with a 1 to 9 bipolar scale with higher scores indicating increasingly negative attitudes (Cronbach’s α = 0.93).

*Harm perceptions*. WTS harm perceptions (perceived harm of WTS relative to cigarettes, perceived likelihood of harm from WTS, and worry about harm from WTS) were assessed using three items: 1) Compared to regular cigarettes, how harmful do you think smoking hookah tobacco is to your health? Response options ranged from 1 (Much less harmful) to 5 (Much more harmful); 2) What do you think would be your chance of getting a serious smoking-related disease, such as cancer, lung disease, or heart disease, if you were to smoke hookah tobacco? Response options ranged from 1 (No chance) to 7 (Certain to happen); and 3) How worried are you about getting a serious smoking-related disease, such as cancer, lung disease, or heart disease, if you were to smoke hookah tobacco? Response options ranged from 1 (Not at all) to 7 (Very much) [23, 37, 38].

*Addiction perceptions*. We assessed addiction perceptions using similar items (i.e., perceived addiction of WTS relative to cigarettes, perceived likelihood of addiction from WTS, and worry about addiction from WTS) with similar response options [23, 37, 38].

*Social aspects of WTS*. We also measured perceived harm of social WTS and flavored WTS using two items: 1) Smoking hookah tobacco for an hour or two in such settings as bars, cafes, and lounges is not harmful to my health and 2) Hookah tobacco that comes in flavors such as menthol or mint, clove, spice, candy, fruit, chocolate, or other sweets is less harmful to my health than unflavored hookah tobacco. Response options ranged from 1 (Strongly agree) to 5 (Strongly disagree). Finally, we measured social acceptability of WTS using the following item: How socially acceptable among your peers do you think each of the following products are? A list of substances followed, including “Hookah.” Response options ranged from 1 (Not acceptable) to 7 (Acceptable).

### Statistical analysis

We used descriptive statistics to characterize the sample and bivariate analyses to assess associations between the extent of susceptibility to WTS and all other study measures using independent samples t-test/one-way ANOVA for categorical variables and Pearson’s correlation for continuous variables. We used multivariable linear regression to examine the association between the extent of susceptibility to WTS and independent variables. For our model building strategy, we built two models, one examining attitudes towards, harms perceptions of, addiction perceptions of, social harm and acceptability of WTS only (Model 1) and another examining sociodemographics and other tobacco and substance use, in addition to all model 1 factors (Model 2). We reported model summary statistics including R^2^ and adjusted R^2^ values, F values, and *β’s* for each independent variable; significance level was set at p < 0.05. Missingness was minimal; therefore, we used complete case analysis. All model assumptions were checked and satisfied, including the linearity assumption using partial regression plots for each continuous predictor; the normality of the residuals assumption using the Q-Q plot; and homoskedasticity using the residuals versus fitted plot. We also assessed for multicollinearity using variance inflation factor (VIF < 10) [39]. All analyses were conducted using SAS 9.4 (Cary, NC).

## Results

### Sample characteristics

A total of 315 participants had never smoked waterpipe and were susceptible with 21 (6.7%) individuals having missing data, leaving a final analytic sample of 294 (**Table 1**). The mean susceptibility score in our sample was 2.1 (SD 0.58, range 1–4). Among susceptible never waterpipe smokers, 59.5% were female, 78.2% attended some college or earned a bachelor’s degree or higher, and 57.8% had an annual household income <$50,000. Approximately two-thirds (68.7%) drank alcohol in the past 30 days, 45.6% used cannabis, 11.2% used e-cigarettes, and 7.5% used cigarillos.

**Table 1:** Study sample characteristics (N = 294).

	N	%	Mean	SD
**Sociodemographics**				
Age			25.0	3.16
Gender	175	59.5		
Female				
Male	119	40.5		
Race Ethnicity				
Non-Hispanic White	113	38.4		
Non-Hispanic Black	62	21.1		
Non-Hispanic Asian	40	13.6		
Non-Hispanic Other	23	7.8		
Hispanic	56	19.1		
Education				
High school or less	64	21.8		
Some college	137	46.6		
Bachelor’s degree or above	93	31.6		
Marital Status				
No	243	82.7		
Yes	51	17.3		
Employment Status				
No	100	34.0		
Yes	194	66.0		
Household Income				
<$50,000	170	57.8		
$50,000 to $99,999	79	26.9		
>$100,000	45	15.3		
**Past-30-day tobacco and substance use**				
Cigarette Use				
No	263	89.5		
Yes	31	10.5		
Cigar Use				
No	278	94.6		
Yes	16	5.4		
Cigarillo Use				
No	272	92.5		
Yes	22	7.5		
Smokeless Tobacco Use				
No	289	98.3		
Yes	5	1.7		
E-cigarette Use				
No	261	88.8		
Yes	33	11.2		
Alcohol Use				
No	92	31.3		
Yes	202	68.7		
Cannabis Use				
No	160	54.4		
Yes	134	45.6		
**Attitudes, perceptions, and social aspects of WTS**				
Susceptibility to WTS (range 1–4)			2.1	0.58
Attitude toward WTS (range 1–9)			6.4	1.81
Perceived harm of WTS relative to cigarettes (range 1–5)			2.9	0.84
Perceived likelihood of harm from WTS (range 1–7)			4.5	1.26
Worry about harm from WTS (range 1–7)			4.6	1.78
Perceived addictiveness of WTS relative to cigarettes (range 1–5)			2.8	0.81
Perceived likelihood of addiction to WTS (range 1–7)			4.1	1.46
Worry about addiction to WTS (range 1–7)			4.0	1.91
Perceived social acceptability of WTS (range 1–7)			3.8	1.66
Perceived social harm of WTS (range 1–5)			2.2	1.02
Perceived harm of flavored WTS (range 1–5)			2.0	0.97

### Bivariate and multivariable analyses

*Sociodemographics*, *other tobacco and substance use*. Consistently in bivariate analysis (**Table 2**) and multivariable analysis (**Table 3 Model 2**), young adults who smoked cigarillos (*β* = 0.53, *p* < 0.01) and used cannabis (*β* = 0.14, *p* = 0.02) in the past 30-days had higher WTS susceptibility. However, using e-cigarettes (*p* = 0.02) in the past 30-days was correlated significantly with WTS susceptibility only in bivariate analysis (Table 2). Additionally, Black non-Hispanic race–ethnicity versus White non-Hispanic young adults (*β* = 0.19, *p* = 0.02) was correlated significantly with WTS susceptibility in multivariable analysis (**Table 3 Model 2**).

**Table 2:** Bivariate associations with susceptibility to waterpipe tobacco smoking (N = 294).

	Mean	SD	r	P-value
**Sociodemographics**				
Age			0.07	0.24
Gender				0.98
Female	2.10	0.57		
Male	2.08	0.58		
Race Ethnicity				0.08
Non-Hispanic White	2.04	0.51		
Non-Hispanic Black	2.23	0.65		
Non-Hispanic Asian	1.98	0.48		
Non-Hispanic Other	2.19	0.61		
Hispanic	2.08	0.62		
Education				0.90
High school or less	2.12	0.65		
Some college	2.08	0.60		
Bachelor’s degree or above	2.09	0.47		
Marital Status				0.83
No	2.10	0.57		
Yes	2.06	0.58		
Employment Status				0.35
No	2.06	0.56		
Yes	2.11	0.58		
Household Income				0.11
<$50,000	2.13	0.57		
$50,000 to $99,999	1.99	0.57		
>$100,000	2.12	0.58		
**Past-30-day tobacco and substance use**				
Cigarette Use				**0.02**
No	2.07	0.55		
Yes	2.31	0.70		
Cigar Use				0.10
No	2.08	0.56		
Yes	2.31	0.68		
Cigarillo Use				**< 0.01**
No	2.04	0.54		
Yes	2.66	0.62		
Smokeless Tobacco Use				0.42
No	2.09	0.57		
Yes	2.30	0.74		
E-cigarette Use				**< 0.01**
No	2.04	0.54		
Yes	2.50	0.67		
Alcohol Use				**< 0.01**
No	1.93	0.57		
Yes	2.17	0.56		
Cannabis Use				**< 0.01**
No	1.97	0.53		
Yes	2.24	0.59		
**Attitudes, perceptions, and social aspects of WTS**				
Attitude toward WTS (range 1–9)			-0.44	**< 0.01**
Perceived harm of WTS relative to cigarettes (range 1–5)			-0.11	0.05
Perceived likelihood of harm from WTS (range 1–7)			-0.22	**< 0.01**
Worry about harm from WTS (range 1–7)			-0.17	**< 0.01**
Perceived addictiveness of WTS relative to cigarettes (range 1–5)			-0.18	**< 0.01**
Perceived likelihood of addiction to WTS (range 1–7)			-0.16	**< 0.01**
Worry about addiction to WTS (range 1–7)			-0.17	**< 0.01**
Perceived social acceptability of WTS (range 1–7)			0.29	**< 0.01**
Perceived social harm of WTS (range 1–5)			0.15	**< 0.01**
Perceived harm of flavored WTS (range 1–5)			0.17	**< 0.01**

Bold values indicate significant results at *p*<0.05.

**Table 3:** Multivariable analysis of correlates of susceptibility to waterpipe tobacco smoking.

		Model 1*			Model 2^†^			
	*β*	95% CI		P-value	*β*	95% CI		P-value
**Sociodemographics**								
Age					0.005	-0.016	0.025	0.66
Gender								
Female					Ref.			
Male					-0.004	-0.118	0.109	0.94
Race Ethnicity								
Non-Hispanic White					Ref.			
Non-Hispanic Black					0.189	0.028	0.350	**0.02**
Non-Hispanic Asian					0.075	-0.107	0.256	0.42
Non-Hispanic Other					0.060	-0.155	0.275	0.58
Hispanic					0.054	-0.103	0.211	0.50
Education								
High school or less					Ref.			
Some college					-0.072	-0.221	0.077	0.35
Bachelor’s degree or above					-0.029	-0.215	0.156	0.76
Marital Status								
No					Ref.			
Yes					-0.044	-0.195	0.107	0.57
Employment Status								
No					Ref.			
Yes					0.043	-0.081	0.168	0.49
Household Income								
<$50,000					Ref.			
$50,000 to $99,999					-0.083	-0.212	0.045	0.20
>$100,000					0.012	-0.161	0.185	0.89
**Past-30-day tobacco and substance use**								
Cigarette Use								
No					Ref.			
Yes					0.088	-0.111	0.286	0.39
Cigar Use								
No					Ref.			
Yes					-0.042	-0.308	0.225	0.76
Cigarillo Use								
No					Ref.			
Yes					0.527	0.303	0.750	**<0.01**
Smokeless Tobacco Use								
No					Ref.			
Yes					0.004	-0.434	0.443	0.98
E-cigarette Use								
No					Ref.			
Yes					0.176	-0.014	0.366	0.07
Alcohol Use								
No					Ref.			
Yes					0.099	-0.025	0.222	0.12
Cannabis Use								
No					Ref.			
Yes					0.136	0.020	0.252	**0.02**
**Attitudes, perceptions, and social aspects of WTS**								
Attitude toward WTS	-0.114	-0.154	-0.074	**<0.01**	-0.080	-0.119	-0.041	**<0.01**
Perceived harm of WTS relative to cigarettes	0.034	-0.048	0.115	0.42	-0.002	-0.076	0.072	0.95
Perceived likelihood of harm from WTS	-0.044	-0.108	0.019	0.17	-0.049	-0.107	0.009	0.10
Worry about harm from WTS	0.026	-0.017	0.070	0.23	0.011	-0.028	0.051	0.58
Perceived addictiveness of WTS relative to cigarettes	-0.067	-0.149	0.016	0.11	-0.084	-0.161	-0.007	**0.03**
Perceived likelihood of addiction to WTS	0.028	-0.028	0.083	0.33	0.026	-0.025	0.077	0.32
Worry about addiction to WTS	-0.028	-0.071	0.014	0.19	-0.011	-0.051	0.028	0.57
Perceived social acceptability of WTS	0.060	0.023	0.097	**<0.01**	0.050	0.015	0.084	**<0.01**
Perceived social harm of WTS	0.005	-0.064	0.074	0.89	0.015	-0.049	0.080	0.64
Perceived harm of flavored WTS	0.013	-0.061	0.087	0.73	0.000	-0.069	0.069	1.00

Bold values indicate significant results at *p*<0.05. *Model 1 included attitudes, perceptions, and social aspects of WTS only. ^†^Model 2 included sociodemographics, other tobacco and substance use, and attitudes, perceptions, and social aspects of WTS

*Attitudes*, *perceptions*, *and social aspects of WTS*. We examined the association of attitudes, perceptions, and social aspects of WTS with susceptibility to WTS using bivariate analysis (**Table 2**) and two multivariable models (**Table 3**). All these factors were significantly associated with susceptibility to WTS except for perceived harm of WTS relative to cigarettes, which was marginally significant (*p* = 0.05). When we examined these factors only (i.e., excluding sociodemographics and other tobacco and substance use) in multivariable analysis (**Table 3 Model 1**), greater susceptibility to WTS was associated only with more positive attitudes towards WTS (*β* = -0.11, *p* < 0.01) and greater perceived social acceptability of WTS (*β* = 0.06, *p* < 0.01). However, in Model 2 (**Table 3**), lower perceived addictiveness of WTS relative to cigarettes (*β* = -0.08, *p* = 0.03) was associated with greater susceptibility to WTS, in addition to the significant correlates found in Model 1 (**Table 3**). Model 2, which included sociodemographics, other tobacco and substance use, and attitudes, perceptions, and social aspects of WTS explained 36.32% of the variance in susceptibility to WTS (R^2^ = 0.3632, Adjusted R^2^ = 0.2932, F = 5.19, *p* < 0.01).

## Discussion

In this study, we examined factors associated with susceptibility to WTS among young adults who had never used waterpipe tobacco. Consistently across models, we found several factors associated with greater susceptibility to WTS uptake, including more positive attitudes towards WTS, lower perceived addictiveness of WTS relative to cigarettes, and greater perceived social acceptability of WTS. Also, our findings indicate that Black non-Hispanic young adults and those who used other tobacco products (cigarillos) or substances (cannabis) were more susceptible to WTS. Existent WTS prevention interventions are mainly risk-based messaging interventions [20, 23, 24]; however, our results indicate that effective WTS prevention interventions should target not only perceived risks of WTS, but also other tobacco and substance use, attitudes, and social acceptability of WTS. These findings can inform the refinement of existing interventions and development of novel interventions targeting young adults who are susceptible to WTS.

We found that Black non-Hispanic young adults appear to be more susceptible to WTS than White non-Hispanic young adults. Moreover, we found that young adults who reported cigarillo or cannabis had higher WTS susceptibility. One prior study examined characteristics of susceptibility to WTS among young adults [20], but it did not examine demographic and other tobacco product or substance use by susceptibility status. Consistent with our findings, another study that investigated openness to use non-cigarette tobacco products found that young adults who used non-cigarette products had higher odds of being open to use waterpipe [22]. Our findings suggests that additional efforts may be needed to prevent WTS in subpopulations at greater risk based on race-ethnicity and other tobacco and substance use. For instance, non-Hispanic Blacks appear to be at greater risk of using flavored tobacco (e.g., menthol cigarettes and cigars) in part due to the deliberate and pervasive targeting by the tobacco industry of black communities [40]. Further research is needed to identify how to target these subpopulations and what factors can be used to target interventions for these subpopulations.

In the two models examining attitudes and perceptions of WTS with and without demographic and behavioral factors, WTS susceptibility to WTS was consistently associated with attitudes towards WTS, perceived addictiveness of WTS relative to cigarettes, and perceived social acceptability of WTS. In line with our findings, Lipkus et. al. investigated characteristics of WTS susceptibility and found that attitudes towards WTS, perceived addictiveness of WTS, and perceived social acceptability of WTS were significantly associated with susceptibility to WTS [20]. However, unlike our findings, that study found additional characteristics that were significantly associated with WTS susceptibility, including relative harm of WTS and risk appraisals (perceived risk and worry about harm and addiction). These differences may be attributed to the varied measurement and study designs. For instance, we used individual measures for perceived harm and addictiveness and risk appraisals while Lipkus et. al. combined harm and addictiveness in a single measure. Although their target population was young adults, they included susceptible and non-susceptible participants who do not smoke waterpipe while we only included those who were susceptible. In a randomized controlled trial, intervention messages focused on targeting young adults’ perceptions of WTS risks, addressing major factors contributing to the appeal of WTS such as flavors and social use [35]. The study indicated noteworthy effects, particularly decreasing initiation among susceptible young adults who do not smoke waterpipe at the highest exposure dose. Moreover, susceptible never smokers demonstrated an increased perception of the addictiveness of WTS two months after exposure to the intervention messages. Consistent with prior studies, our findings emphasize the importance of influencing attitudes towards, perceived addictiveness of, and perceived social acceptability of WTS to reduce the susceptibility to WTS among young adults who do not smoke waterpipe, and, potentially, reduce WTS uptake.

Many young adults are unaware of WTS risks and misperceive it as a less harmful product than other tobacco products, such as cigarettes [41–43]. Our bivariate analysis indicated the importance of WTS health harm perceptions among susceptible young adults who do not smoke waterpipe. However, this association was attenuated in the multivariable models. Extant interventions targeting WTS prevention are mainly risk-based messages, which were successful in raising harm beliefs and reducing susceptibility to future WTS [20, 23, 24, 35]. In one recent study, researchers examined the effect of WTS risk messages on WTS risk appraisals, attitudes towards WTS, ambivalence about WTS, and willingness to smoke waterpipe in young adults aged 18–30 years [37]. The study revealed that the risk messaging intervention heightened risk appraisals, which in turn induced more negative attitudes towards WTS, leading to reduced willingness to WTS. This mediation analysis aligns with our results by indicating that attitudes may be a significant mechanism through which risk messaging impacts susceptibility to WTS, and in turn waterpipe use behavior.

Our findings—in line with successful interventions that have effectively heightened the perceived addictiveness of WTS among young adults [35]—also extend our understanding of young adults’ susceptibility to WTS, underscoring the importance of addressing perceived addictiveness in interventions to prevent WTS. The belief that WTS is less addictive than cigarettes—a common misperception among young adults—represents an opportunity for interventions to potentially decrease susceptibility [8, 9, 25, 44, 45]. However, little information exists regarding addiction beliefs relevant to WTS among susceptible young adults who do not smoke waterpipe to reduce susceptibility to WTS uptake. This information is crucial for the development of targeted interventions with the goal of diminishing susceptibility and preventing the initiation of WTS among this population. Expanding our understanding of specific beliefs associated with addiction in the context of WTS among susceptible young adults who do not smoke waterpipe will contribute valuable insights to the design and implementation of more effective prevention strategies.

Our study had notable strengths, such as using a sample from a nationally representative consumer research panel and using literature to guide our variable selection and analysis. Also, the measures we used in our study were validated in young adults. However, there were some limitations to our study results. Our analysis was cross-sectional, so we cannot establish the temporality between correlates and susceptibility to WTS among young adults. Although we recruited participants from a nationally representative consumer research panel, restricting our sample to susceptible young adults who do not smoke waterpipe may limit the generalizability of our findings to other populations. Larger, more representative, longitudinal studies are needed to replicate our findings. Additionally, several factors were unmeasured, including exposure to marketing strategies and advertising targeting young adults, exposure to secondhand smoke at home and social settings, parental smoking history, and perceived social benefits of smoking, which may influence susceptibility to WTS.

## Conclusion

This study identified several potentially modifiable correlates of susceptibility to WTS, which can inform which factors may be best to target in WTS prevention intervention among young adult susceptible to WTS. Consistently across models, our analyses indicated that several factors were associated with susceptibility to WTS uptake, including attitudes towards WTS, perceived addictiveness of WTS relative to cigarettes, and perceived social acceptability of WTS. Additionally, our findings suggested demographic and behavioral differences, including racial and ethnic (Black non-Hispanic) and other substance use (cigarillo, cannabis) differences. These results could assist in improving current interventions and creating new interventions for young adults who do not smoke waterpipe susceptible to WTS, and subsequently reduce WTS uptake in this population. Longitudinal studies are also needed to better understand the role of attitudes and perceptions in WTS susceptibility and what specific beliefs are relevant to those factors that contribute to WTS susceptibility.
